# Functional Analysis: Evaluation of Response Intensities - Tailoring ANOVA for Lists of Expression Subsets

**DOI:** 10.1186/1471-2105-11-510

**Published:** 2010-10-13

**Authors:** Fabrice Berger, Bertrand De Meulder, Anthoula Gaigneaux, Sophie Depiereux, Eric Bareke, Michael Pierre, Benoît De Hertogh, Mauro Delorenzi, Eric Depiereux

**Affiliations:** 1 Bioinformatics and Biostatistics Laboratory, Molecular Biology Research Unit (URBM), FUNDP University of Namur, Namur, Belgium; 2 Swiss Institute of Bioinformatics, Lausanne, Switzerland

## Abstract

**Background:**

Microarray data is frequently used to characterize the expression profile of a whole genome and to compare the characteristics of that genome under several conditions. Geneset analysis methods have been described previously to analyze the expression values of several genes related by known biological criteria (metabolic pathway, pathology signature, co-regulation by a common factor, etc.) at the same time and the cost of these methods allows for the use of more values to help discover the underlying biological mechanisms.

**Results:**

As several methods assume different null hypotheses, we propose to reformulate the main question that biologists seek to answer. To determine which genesets are associated with expression values that differ between two experiments, we focused on three ad hoc criteria: expression levels, the direction of individual gene expression changes (up or down regulation), and correlations between genes. We introduce the FAERI methodology, tailored from a two-way ANOVA to examine these criteria. The significance of the results was evaluated according to the self-contained null hypothesis, using label sampling or by inferring the null distribution from normally distributed random data. Evaluations performed on simulated data revealed that FAERI outperforms currently available methods for each type of set tested. We then applied the FAERI method to analyze three real-world datasets on hypoxia response. FAERI was able to detect more genesets than other methodologies, and the genesets selected were coherent with current knowledge of cellular response to hypoxia. Moreover, the genesets selected by FAERI were confirmed when the analysis was repeated on two additional related datasets.

**Conclusions:**

The expression values of genesets are associated with several biological effects. The underlying mathematical structure of the genesets allows for analysis of data from several genes at the same time. Focusing on expression levels, the direction of the expression changes, and correlations, we showed that two-step data reduction allowed us to significantly improve the performance of geneset analysis using a modified two-way ANOVA procedure, and to detect genesets that current methods fail to detect.

## Background

### Introduction

The major issue when studying datasets with many tests and few replicates in general and microarray datasets in particular is the decreased power of the analysis. Indeed, to reduce the risk of detecting false positives due to the lack of a sufficient number of replicates, statistical tests allow for higher threshold levels as the number of replicates decreases. This implies an unavoidable increase of the number of false negatives and, thereby, a decrease in power.

To compensate for this lack of information about expression data generated by microarrays, some authors have tried to work at the probe level, breaking down probesets into smaller components. Nonetheless, most researchers prefer to work at the probeset level where data can be analyzed in different layers (individual analysis, geneset analysis, coexpression studies, clustering) [1].

Among these methods, the analysis of genesets with regard to known or observed biological criteria holds high potential for fundamental as well as clinical research. For example, members of the same metabolic pathway can be studied simultaneously to assess modifications of the pathway under certain conditions, such as the action of a drug, or to diagnose a pathology, etc. [2]. This last application is common with genesets called "signatures", devised for certain pathologies based on coexpression [3] or clustering studies [4,5]. The study of genesets involves two complementary tasks:

- group discovery: empirical data is used to identify new criteria to discover new genesets. This task uses several different approaches such as data clustering [6-9] and the study of regulatory sequences [10-14],

- geneset analysis: previously defined genesets are used to guide empirical data analysis, to determine whether the expression values of known genesets are correlated with the conditions compared in the experiments [15-17].

The research presented here focuses on statistical geneset analyses. Our initial motivation was to answer the seemingly trivial question (Q_0_): "Which known genesets are associated with different expression profiles under the two conditions compared?". We discuss here the ability of current methods to provide an answer to this question.

This main question Q_0 _implies several hypotheses related to the nature/composition of genesets tested: the individual members (genes) may be expressed at various levels, over- or under-expressed or correlated (coordinated response of member genes).

To provide an answer to question Q_0_, we present the FAERI methodology (Functional Analysis: Evaluation of Response Intensities), an ANOVA-2-like procedure tailored to detect genesets with differing expression profiles, regardless of the nature of the individual responses.

### State of the art

Several geneset analysis methods have been published recently. Each available method provides an answer to question Q_0_. The methods discussed below were selected to provide an overview of the main strategies followed by the authors, or due to their pioneering approach.

Over-representation analysis methods are not considered in this paper, as they focus on the top list obtained by individual analysis and aim to identify only those groups of genes that are represented by the most differentially expressed probesets. This approach thus facilitates the interpretation of individual analyses, by giving annotation clues about biological meaning. However, such methods require the ad hoc selection of an individual statistic, and the definition of an arbitrary threshold for the detection of individual genes, whatever the individual statistic used. Thus, over-representation analysis does not allow for the detection of genesets associated with subtle individual changes according to the individual statistic chosen [18-21]. For this reason, other authors describe improved ORA strategies. As an example, Yi and Stephens, in SLEPR, first select sample-level differentiated genes from each individual sample, before computing an enrichment score characterizing the geneset [22].

Table 1 contains several questions addressed by previously described methods.

**Table 1:** Formulation of the different questions asked by the different genesets analysis methods

Question	Formulation
Q_0_	Which known genesets are associated with different expression profiles under the two conditions compared?

Q_comp_	Which geneset definitions are associated with the biggest difference in expression profiles observed under each condition?

Q_self_	Which genesets are associated with diverging expression profiles between conditions compared with random definitions of phenotype?

Q_cor_	Which genesets are defined by members associated with correlated expression changes?

Q_uni_	Which genesets are associated with an increase or decrease of all member expression values?

Q_bidir_	Which genesets are defined by differentially expressed genes, regardless of the direction of the regulation?

Q_int_	Which genesets are associated with variable individual expression changes?

Q_u_^c^	Which unidirectional groups have members that are associated with a correlated answer?


Question Q_0 _covers all the others; therefore, a method answering this question answers all the others.

More recent functional class scoring (FCS) methods can be classified based on their competitive or self-contained null hypothesis. Competitive methods sort the groups focusing on question Q_comp _(Table 1) [23-28]. Such methods provide a list of genesets that are the most significantly associated with the phenotype compared with other geneset definitions. The definition of the null hypothesis of the competitive methods is theoretically associated with the gene-sampling strategy for significance evaluation (data permutations driven by random definition of equal sized genesets) [23,29]. This statement, however, as shown by Effron and Tibshirani, does not take into account the pattern of correlations between genes, which is destroyed by gene-sampling [29]. Conversely, self-contained methods study question Q_self _(Table 1). The significance of the results, for those methods, is evaluated by label sampling (data permutations driven by random definition of phenotypes for a given group definition). Each geneset score is thus evaluated against its putative range of geneset score values, maintaining the geneset definition [23,28,30-34].

The GSEA methodology and its derivatives make use of a hybrid strategy providing results that are harder to interpret. The null model used to develop the methods relies on the competitive approach, but is later evaluated by label sampling, thus mixing both strategies [24,25,27].

The second methodological difference between the methods concerns definition of the geneset score evaluated. Methods using two steps first compute an individual statistic that is then used in a second step to compute a geneset statistic [23-28,33]. Conversely, global methods perform the analysis in a single step, evaluating geneset statistics from raw expression values associated with the group members (GlobalTest, GlobalAncova) [30,31,34]. SAMGS is a particular case of a global method where the final formulation of the statistic is similar to a two-step strategy using the SAM t-like individual statistic [30].

### Questions studied by the methods

Current methods do not provide equivalent answers to question Q_0_. Therefore, we propose to reformulate the hypothesis used with the available methods with respect to the biological properties of the sets (Table 1).

Table 2 provides a snapshot of several available analytical methods. These methods are compared based on the hypothesis tested (3^rd ^column), data used (4^th ^column), statistic used (5^th ^column) and significance evaluation procedure (6^th ^column). The other columns provide additional information.

**Table 2:** Comparison of the properties of several geneset differential expression analysis methods

Author	Year	Hypothesis	Data used	Group statistic	Significance	Name	Properties	Individual statistic
**Mootha et al**	**2003**	**Competitive**	**Individual statistic**	**ES (Running Sum)**	**Sample permutations**	**GSEA**	**Hybrid**	**Signal/noise Ratio**
**Subramanian et al**	**2005**	**Competitive**	**Individual statistic**	**ES (Running Sum)**	**Sample permutations**	**GSEA**	**Hybrid, asymmetrical**	**Individual correlation (r)**
**Keller et al**	**2007**	**Competitive**	**Individual statistic**	**ES (Running Sum)**	**Competitive theoretical model**	**Variant GSEA**	**Hybrid, symmetrical**	*
**Effron & Tibshirani**	**2007**	**Competitive**	**Individual statistic**	**ES (Running Sum)**	**Sample permutations**	**GSA**	**Restandardizaton**	*
**Pavlidis et al**	**2004**	**Competitive**	**Individual statistic**	**Log (p(g)) = mean[log[p(i)]]**	**Genes permutations**		**Depends on the geneset size**	**Pearson correlation coef**.
**Tian et al**	**2005**	**Competitive**	**Individual statistic**	**Weighted mean**	**Genes permutations**		**Standardization**	**Student t**

**Tian et al**	**2005**	**Self-contained**	**Individual statistic**	**Weighted mean**	**Sample permutations**		**Standardization**	**Student t**
**Kim & Volsky**	**2005**	**Self-contained**	**Individual statistic**	**Mean**	**Normal distribution**	**PAGE**	**Central Limit Theorem**	**Fold-change**
**Effron & Tibshirani**	**2007**	**Self-contained**	**Individual statistic**	**Mean**	**Sample permutations**	**GSA**	**Unidirectional** **+ Restandardization**	**Student t**
**Effron & Tibshirani**	**2007**	**Self-contained**	**Individual statistic**	**Maxmean**	**Sample permutations**	**GSA**	**Unidirectional (directional subset) + Restandardization**	**Student t**
**Effron & Tibshirani**	**2007**	**Self-contained**	**Individual statistic**	**Absmean**	**Sample permutations**	**GSA**	**Unidirectional (absolute value) + Restandardization**	**Student t**
Dinu et al	2007	Self-contained	Expression data	Sum (d^2^)	Sample permutations	SAM-GS	Determination of S_0_	SAM d statistic
Goeman et al	2004	Self-contained	Expression data	Q(g) = mean(Q(i))	Permutation/Gamma/Asymptotic	GlobalTest	P (Y|X)	Q(i)
Mansmann & Meister	2005	Self-contained	Expression data	F	Sample permutations	GlobalAncova	P (X|Y)	
*Berger et al*	*Unpublished*	*Self-contained*	*Expression data*	*F*	*Fisher F*	*ANOVA-2*	*Unidirectional*	
*Berger et al*	*Unpublished*	*Self-contained*	*Expression data*	F*	*Sample permutations/random data*	*FAERI*	*Bidirectional*	

The methods are grouped into categories. The upper and lower parts of the table list respectively the competitive and self-contained methods. The methods highlighted in bold rely on a two-step procedure. The methods in plain writing rely on a global analysis, which uses the expression data to compute the geneset statistic in one single step, based on multivariate models. Finally, ANOVA-2 and FAERI are shown in italics.

Question Q_0 _includes all of the other questions (Q_cor_, Q_uni_, Q_bidir _and Q_int_) related to the nature of the groups tested. Three criteria must be taken into account to describe the groups: expression levels (a), the direction of the expression change (b) and correlations between the group members (c).

(a) It is important to study expression levels as highly expressed probesets may mask differences observed for probesets with lower expression levels. Two-step methods such as the SAMGS are not influenced by this criterion. For example, the Student *t *or SAMGS *d *statistics characterize expression differences, regardless of the individual expression levels [30]. Moreover, global methods that rely on the i.i.d. condition may be affected as expression levels vary between probesets, meaning that the probesets are not identically distributed.

(b) Individual expression values may reveal up or down regulation of the genes depending on the conditions tested. One way to take the direction of expression changes into account and deliver results with no dependence on this criterion is to avoid studying mixed groups with a global null effect (50% over and 50% under expressed). Two-step methods may be adapted to use the absolute value of a unidirectional individual statistic (Student *t *[35]), or its squared value (SAMGS [30]). Alternatively, the geneset statistic may be defined to be representative of the directional subgroup associated with the biggest expression change (absmean), optionally with regard to the size of the directional subgroup (maxmean) [23]. Among global methods, the SAMGS and GlobalTest are independent of the direction as the null hypothesis is tested respectively on squared statistic (*d^2^*) and expression change variability (*τ^2^*) [30,31].

(c) Genes may be associated with correlated or variable expression changes. Question Q_0 _takes both possibilities into account. Two-step methods based on a sum or a mean, like the SAMGS, do not need further adaptation since they explicitly rely on the sum of individual pieces of information [5,23,28,30]. The total difference is then evaluated independently of the variability of the individual differences. Among global methods, the GlobalTest is suited for the study of uncorrelated genesets as it assesses the variability of the individual answers. On the contrary, the GlobalTest is not intended to detect correlated groups since the variability of the individual answers in such sets is null (question Q_u_^C^, see Table 1) [31].

It appears that only two-step methods, as well as the SAMGS, may simultaneously provide an answer to the three criteria considered and thus evaluate more completely question Q_0_.

Software packages developed thus far are not always able to fine-tune the appropriate combination of steps with regards to the criteria. Moreover, it is not systematic to take the direction of the answer into account or to rely on partial genesets (the maxmean statistic, for example, mainly takes the directional subgroup with the strongest expression change into account) [23,28,30,33].

Global methods, on the other hand, ignore the possible bias associated with expression level. The SAMGS procedure involves a preliminary step of variance stabilization based on the SAM individual analysis method, which requires that the expression values of all probesets be estimated [30,36-38].

Competitive procedures aim to identify groups with bigger differences and ignore groups associated with the phenotype but with a moderate answer compared to the others. The self-contained null hypothesis thus provides a more appropriate answer, where each set is evaluated according to its possible distribution range [23-28,33].

### Objectives

There are several possible strategies to evaluate question Q_0 _as completely as possible. Several methodologies may be selected to answer complementary questions. Gentleman et al. previously suggested to separately study sub-groups defined by the direction of expression changes and to define core sets of genes common to several genesets (thus avoiding that the same genes lead to the detection of several genesets). As a perspective of the GlobalAncova methodology, Mansmann and Meister proposed to study the interaction between the effect of the condition and group composition, to take the variability of the individual responses into account. The hypothesis associated with this test complements the unidirectional null hypothesis, but also provides a partial answer to question Q_0 _since it ignores unidirectional correlated groups (no interaction).

The work reported below proposes an alternative strategy to answer question Q_0 _based on a single self-contained test. To optimally analyze expression data, our model relies on a global strategy because two-step methods suffer from a loss of information (correlation) caused by the substitution of data with individual statistics. We introduce the FAERI method that was tailored from the 2-factor ANOVA procedure. Our aim was to group the information associated with individual differences into one single statistic taking direction, correlation and expression level into account and to combine the advantages of the GlobalTest (groups with a variable answer), GlobalAncova (unidirectional groups) and SAMGS (independence with expression level) models whilst avoiding their respective limitations.

## Methods

### Datasets

#### Hypoxia datasets

Bosco et al. (2006) described the E-MEXP-445 dataset, available in the ArrayExpress repository. The experiment compares samples extracted from human monocytes. RNA was hybridized on 6 Affymetrix HGU-133a microchips, among which 3 normoxia samples and 3 samples from cultures grown under hypoxic conditions [39].

Vengellur et al. (2005) studied cellular response to hypoxia on human Hep3b hepatocytes cell lines, considering 3 additional treatments known to mimic hypoxic conditions: cobalt chloride (100 μM), nickel chloride (100 μM), and DFO (100 μM). mRNA was extracted separately from two biological replicates, and cRNA was hybridized on Affymetrix GeneChip HG-U95Av1 microchips. We used part of this dataset to compare samples grown under normoxic and hypoxic conditions, and did not use the other samples (used by the authors to compare hypoxia with molecules that mimic hypoxic conditions). This dataset is available on GEO (GSE-1056) [40].

Kim et al. (2006) described the GSE-4086 dataset, available on GEO. The authors studied cellular response to hypoxia on human B lymphocytes (P493-6 cell line). Two biological replicates were used to extract mRNA from separate cultures. cRNA was hybridized on Affymetrix GeneChip HG-U133A microchips [41].

In the work reported here, the three hypoxia datasets were preprocessed using GCRMA to study the effect of hypoxia and compare the ability of each geneset analysis method to detect common pathways from several datasets featuring a limited number of replicates.

Xiao et al. (2008) report a study of osteoporosis using microarrays hybridized with 10 low and 10 high BMD samples (post-menopausal woman, aged 54-60). B-lymphocyte mRNAs were isolated from 70 ml of blood, and cRNA was hybridized on Affymetrix GeneChip HG-U133A microchips. This dataset is available both on GEO (GSE-7429) and ArrayExpress (E-GEOD-7429) [42]. We used this dataset, preprocessed with GCRMA, to illustrate the development of the FAERI methodology.

### Introduction to FAERI

As several genes are associated with multiple probesets, a bias could be introduced during the analysis at the geneset-level. In the work reported below, we used only one probeset for each gene studied on the microchip, by selecting the probeset associated with the largest variability, to avoid giving too much weight to a gene due to the number of associated probesets. As a result of this selection, the words "probesets" and "genes" are synonyms in this text. The multiplicity of the probesets is not considered in this publication.

The analysis of variance or ANOVA relies on the study of the variability of the data with respect to one or more criteria to determine if that(those) criterion(a) is(are) responsible for the values observed. The simplest model, relying on a single criterion is equivalent to the Student *t *test [43]. When analyzing differential expression, a one-way ANOVA can evaluate the involvement of probesets under two or more conditions. The ANOVA with two classification criteria allows for study of the differential expression of a set of probesets (the first and second criteria respectively relate to the members of the geneset and to the definition of the experimental conditions compared).

The model that we chose to test the validity of this approach states that the expression data observed can be explained by dependence of the intensities measured on the probesets, the condition and the interdependence between those two parameters, since each probeset can potentially be associated with a different response under each of the tested conditions.

The mathematical representation of this model is given by Equation 1.

(1) $$X_{(ij)k}=\mu +a_{i}+b_{j}+ab_{ij}+E_{(ij)k}$$

Where μ is the general mean, a_i _is the contribution of factor a, b_j _is the contribution of factor b, ab_ij _is the contribution of the interaction of factors a and b and E_(ij)k _is the residual contribution.

The comparison between the different criteria relies on the ratio of the mean squares associated with each criterion. The statistic computed for each criterion is evaluated by Fisher's F distribution using the appropriate number of degrees of freedom (equations 2 to 4).

(2) $$F_{a}=\frac{MS_{a}}{MS_{E}}\sim F(n_{a}-1;n_{a}n_{b}(n_{repl}-1))$$

(3) $$F_{b}=\frac{MS_{b}}{MS_{E}}\sim F(n_{b}-1;n_{a}n_{b}(n_{repl}-1))$$

(4) $$F_{ab}=\frac{MS_{ab}}{MS_{E}}\sim F(n_{a}-1)(n_{b}-1);n_{a}n_{b}(n_{repl}-1))$$

Where F_x _is the F score associated with factor x, MS_x _is the mean square value associated with factor x, n_x _is the number of levels for factor x.

It is important to note that when the effect of the interaction is significant, no decisions can be made for the other criteria. The study of the effect associated with the condition is close to the GlobalAncova method [31,44]. The study of the interaction is similar to the GlobalTest methodology and to the perspectives formulated by Mansmann and Meister (GlobalAncova), since the genesets within which probesets present a different answer are biologically interesting [44]. We state that this test must be complemented with a test on the condition because it is impossible to detect unidirectional and correlated changes by studying the interaction alone. To circumvent this limitation, we present a strategy in the next section for analysis of the FAERI methodology designed to simultaneously answer all possible configurations in a single test. For more details on the procedures of the FAERI methodology, see the Additional File 1 (Section 1).

### FAERI

Geneset analysis of differential expression is influenced by the direction of the expression changes of the members and by possible interactions between the probesets and the conditions [29,31,44-46].

#### Expression level

Probesets belonging to the same geneset can be expressed at various levels. From a biological point of view, a weakly expressed geneset might have a bigger impact on physiology than a strongly expressed one. We propose to standardize the data so that, in principle, all the probesets have the same potential for response under two conditions compared, based on a classical reduction of data producing Z values. The standardization is computed for each probeset using Equation 5 by pooling the data associated with both conditions and computing the observed mean and variance. The resulting reduced data is distributed around a mean value of 0 with a variance of 1. The information about the difference between the conditions is retained. At the end of this operation, the result of an individual analysis based on the Student *t *is unchanged. Note that this procedure renders data closer to the conditions of application of the ANOVA 2 (iid values).

(5) $$Z_{ij}=\frac{\left(X_{ij}-M_{pool,i}\right)}{S_{pool,i}}$$

Where X_ij _is the expression value associated with the i^th ^probeset in the j^th ^microchip, M_pool, i _and S_pool, i _are the means and standard deviations computed for probeset i from all experiments (step 1). This procedure is repeated for each probeset in the dataset.

#### Direction of differential expression

Several probesets may be activated or repressed at the same time, but may also be associated with a variable expression change, depending on the biological context studied and the definition of the genesets. To provide a full answer to question Q_0_, with no dependence on the direction of differential expression, two approaches can be proposed: up- and down-regulated probesets may be studied separately or simultaneously by computing a cumulative value for the absolute difference. The first solution consists of defining "activated" or "repressed" genesets to conduct a proper analysis. This approach relies on current knowledge that is incomplete, but is useful for diagnosis purposes to determine whether a patient has a pathology by analyzing signature genesets. The second solution is addressed by several methods by defining an individual statistic independent of the direction (absolute or squared value of the Student *t *statistic) [23,37,47,48].

Using a multivariate procedure, we propose to study the absolute response of the genes by directional reduction of the data. This operation involves multiplying the expression values related to down-regulated genes by -1 (Equation 6). The down-regulated genes are identified empirically from the expression data studied.

(6) $$Z_{ij}^{D}=sign(D_{i})\times Z_{ij}$$

Where D_i _= M_i_^B ^- M_i_^A^, the difference in means between conditions A and B for the i^th ^probeset, Z_ij _= Z-score associated with the i^th ^probeset in the j^th ^microchip, and Z^D^_ij _the directionally reduced Z-score associated with the i^th ^probeset in the j^th ^microchip.

It may be interesting to consider the median instead of the mean to evaluate the direction of differential expression. However, the performances tests we conducted previously in individual expression analyses show a reduction in the performance of the Student *t *test when using the median as the estimator [49] on biological data (and on spike in datasets, data not shown).

After this 2-step reduction of the data, FAERI evaluates an F statistic as is done in the ANOVA-2 procedure (Equation 1). The FAERI F* statistic associated with the condition effect is representative of the differences found based on biological reasoning. Each gene provides information on its differential expression, independently of the direction of that difference, and the absolute value of the individual *t *statistic remains unchanged.

### Null distribution

Due to the dependence on geneset size introduced by directional reduction, the significance of the results cannot be evaluated using Fisher's theoretical distribution, which is used by the ANOVA procedure. We will refer to the statistic computed using the FAERI methodology as F*. We tested two solutions to determine the null distribution of the FAERI F* statistic, relying either on permutations or the use of random data.

The permutation strategy associated with a self-contained null hypothesis relies on label sampling [23,29]. Given the null hypothesis that no probeset is involved under the conditions compared, the permutations of the labels are performed independently for each probeset, independently of the correlation between the members of the geneset.

Due to the Z value reduction step, a second strategy may be used to evaluate the null distribution of the FAERI F* statistic. Indeed, as the null hypothesis states that there are no differences between the two conditions compared and as we postulate that individual expression values follow a normal distribution, Z standardization provides the possibility to establish the null distribution using random data from a normal distribution, and then apply the same 2-step reduction to compute the reference F*_0 _values. Under the null hypothesis, the F* statistic computed on reduced test data should follow the same distribution as the F*_0 _statistic computed from reduced random data. Conversely, if the expression values of the geneset differ between two conditions, the F* statistic distribution leads to higher values than the F*_0 _values computed from random data.

This possibility to compute a reference distribution based on random data frees the FAERI method from the limited number of available permutations. Moreover, for each experimental strategy (number of replicates) and each geneset size, the null distribution can be computed once and stored for future analysis.

## Results and Discussion

### The effects of two-step reduction

**Figure 1:**
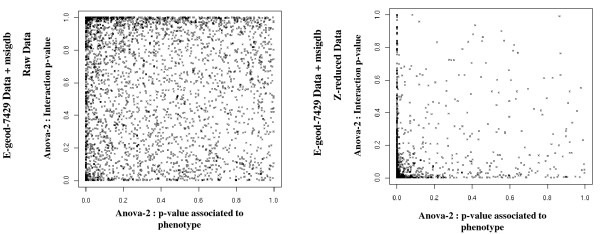
**Illustration of the effect of Z-value data reduction on a variance analysis with two classification criteria**. The p-values associated with the effect of the condition studied and the intersection are compared before and after Z reduction. After this step, the effect associated with the probeset is null (data not shown). Prior to Z reduction, the probesets are expressed at variable levels. After reduction, the expression level is standardized for all of the probesets and their individual contributions are balanced during the variance analysis. The genesets analyzed are distributed differently and reveal a more pronounced effect of the condition and/or of the interaction between the condition and the probesets. Put differently, both the strength and variability of the individual answer are revealed by this step.

As presented in the introduction, we propose to take the level of individual expression into account, using a first-step reduction of the data. Figure 1 illustrates the results obtained when applying ANOVA-2 to the raw data and to the reduced data. In both cases, the effect of the condition factor was compared to the interaction between the condition and the probeset. Comparison of the p-values associated with the two effects, before and after standardization, shows that expression levels are an important criterion: a very large number of groups are significant for one of the two effects or for both when each probeset is considered on even ground. This suggests that a very large number of genesets are made up of members distributed at various expression levels and that the expression change of several weakly expressed probesets is masked by strongly expressed probesets when raw data is used.

In the second step of FAERI, we used a directional reduction to obtain a cumulated value of absolute expression changes in the genesets. We chose to use an empirical determination of the direction, easier to assess from the data than from prior incomplete knowledge. As the sign of the reduced values is not a feature of the biological mechanisms involved within genesets, the description of the mechanisms involved should be associated with individual analysis of the member genes based on raw data.

**Figure 2:**
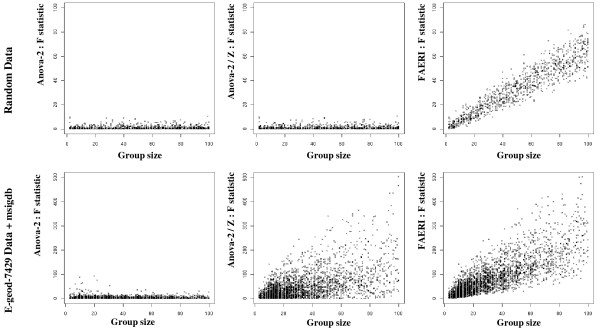
**Illustration of the distribution of the F statistic evaluated compared with geneset size using the ANOVA-2 procedure on the initial expression data (left panel), on the standardized data (center) and on standardized and unidirectional data (right, FAERI procedure)**. The graphs in the upper part are generated from random data and show that the directional reduction step induces dependence on the number of members in the geneset. The graphs in the lower part show results obtained from real data (E-GEOD-7479), and illustrate the impact of the standardization of data relative to each probeset as well as dependence on the number of members following the directional reduction step.

Figure 2 illustrates the distribution of the F* statistic with regards to the number of members, for each step of the FAERI analysis strategy. The upper part of the figure shows the results obtained with randomly generated data and the lower part shows results obtained with the biological dataset E-GEOD-7429. The statistics computed on real data are larger, which highlights the important biological variability. The Z reduction step considerably modifies the distribution of the F* values on the biological dataset, thus underscoring the effect associated with expression level as previously mentioned and observed in figure 1. This step thus has a major impact on the results of the analysis. Directional reduction leads to dependence on the number of members in the group. Random sampling of a small number of individual values leads to a bias associated with individual variability. On average, this bias is equally probable in both directions. Therefore, applying an ANOVA-2 analysis to such data reveals that the mean effect is null. After directional reduction of the data, the global effect of this bias is cumulated and added to the effect associated with the condition. Since this bias is cumulated, the F* statistic is dependent on geneset size and increases when the set has more members. This concern can be assessed quite easily as described in the "Materials and Methods" section of this paper, through suitable evaluation of the null distribution.

**Figure 3:**
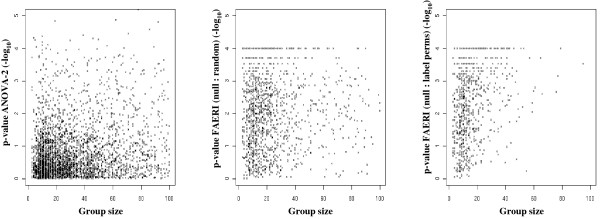
**Illustration of the logarithm of the p-values obtained by ANOVA-2 (left), FAERI based on random data (center) or permutations (right), versus the number of members in the geneset (real dataset E-GEOD-7479)**. The graphs presented in the center and on the right show that the two procedures to evaluate the significance of the FAERI test give p-values dependant on geneset size.

**Figure 4:**
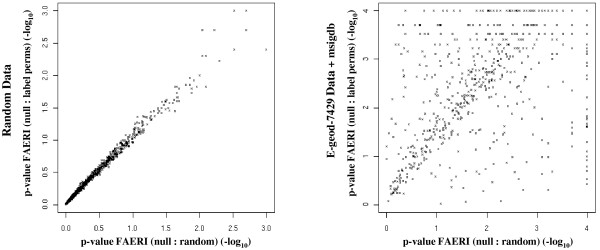
**Comparison of the logarithm of the p-values obtained by FAERI based on random data or permutations**. The left graph shows the comparison of the p-values obtained during analysis of simulated data. The right graph shows results obtained when analyzing real data (E-GEOD-7479), illustrating that the null distribution evaluated by the two procedures is different in the case of real data, but, nonetheless, that part of the genesets present a similar p-value (diagonally).

We tested two procedures to assess the significance of the F* statistic. Figure 3 shows that the computed p-value is independent of the number of members in the set, regardless of the procedure used. Figure 4 shows that the p-values evaluated using permutations or random data are perfectly correlated when simulated data is analyzed, but that a great number of genesets display different behavior when biological data is analyzed. This observation illustrates that the variability of biological data cannot be modeled by generating a null distribution from iid random data, as both procedures do not provide similar p-values when real-world data is analyzed. In biological samples, each gene may be expressed at a variable level of expression, with gene-specific variability. Furthermore, a correlation exists between genes, so that their distribution is not independent. The evaluation of permutations leads to a higher number of extreme values, as co-occurring expression changes are more likely to be observed using biological data.

Mansmann and Meister recommend assessing the significance from permutations instead of the Fisher distribution when analyzing microarray data with a multivariate procedure. Their GlobalAncova procedure evaluates the significance of the test using the label-sampling strategy instead of the classic Fisher distribution [34]. The GlobalAncova procedure does not include a Z-reduction step, and the iid requirement is not met due to gene-specific expression levels, gene-specific variability, and gene correlation patterns.

### Performance evaluation

#### Performance evaluation on simulated data

Several authors have stressed the need to compare the performances of geneset analysis methods with regard to the correlation between geneset members [23,29,50]. To this end, Marit Ackermann et al. developed a simulation scenario focusing on 9 geneset definitions [51]. Each set is generated randomly by a multivariate normal distribution using the definition of a correlation/covariance matrix. Table 3 summarizes the parameters used to fine-tune the simulation of the sets, focusing on the correlation structure, direction of simulated differences, and proportion of members differentially simulated. Each set was simulated with 10 replicates for each condition and included 20 members.

**Table 3:** Definition of the series of measurements used to evaluate the performances of the geneset differential expression analysis methods

	Difference of expression	Correlation	Diff. Expressed	Over-Expressed	Under-Expressed	Design
Set 1	0.75	0.6	20	20	0	Unidirectional

Set 2	0.75	0	20	20	0	Unidirectional

Set 3	0	0	0	0	0	H_0_

Set 4	0.75	0.6	10	10	0	Unidirectional

Set 5	0.75	0	10	10	0	Unidirectional

Set 6	1	0.6	20	10	10	Bidirectional

Set 7	1	0	20	10	10	Bidirectional

Set 8	1	0.6	10	5	5	Bidirectional

Set 9	1	0	10	5	5	Bidirectional

We reproduced the simulation proposed by Ackerman et al. to assess the performances of ANOVA-2, FAERI and several geneset analysis methods currently available, to obtain a comprehensive understanding of the performances with regards to the mathematical structure of the data. Each set was simulated 100 times and Table 4 summarizes the detection rate associated with each set, when the selection threshold was set to 0.1, 0.01 and 0.001 for the p-values. Histograms of the p-values resulting from the analysis of set no. 3 with each method are also provided in the Additional File 1.

**Table 4:** Comparison of geneset differential expression analysis method performances, based on the simulation model proposed by M Ackerman

		Cut-Off		a2.fixed	faeri. fixed.null	faeri. fixed.perms	GSA. mean*	GSA. absmean*	GSA. maxmean*	Globaltest. asymptotic	Globaltest. gamma	Globaltest. permutations	Gsea. pval	Gsea. fdr	Samgs. pval	Samgs. qval
		**0.001**	**Set3**	0	0	0	0	0	0	0	0	0	0	0	0	1
**H_0_**		**0.01**	**Set3**	1	0	0	0	0	0	0	0	0	0	0	0	2
		**0.1**	**Set3**	3	3	3	12	0	0	2	5	3	2	1	2	27

		**0.001**	**Set2**	100	96	97	15	25	12	54	96	95	69	20	91	94
	100% DE	**0.01**	**Set2**	100	99	99	15	25	12	95	99	97	99	77	98	99
**Uni**		**0.1**	**Set2**	100	100	100	78	72	64	99	99	99	100	100	99	100
	
		**0.001**	**Set5**	72	40	36	0	2	0	3	64	49	14	2	39	56
	50% DE	**0.01**	**Set5**	89	75	78	0	2	0	42	89	84	42	25	81	84
		**0.1**	**Set5**	100	94	94	7	17	3	93	99	98	93	84	96	99

		**0.001**	**Set1**	86	62	60	1	2	2	1	10	8	2	0	7	13
	100% DE	**0.01**	**Set1**	89	69	70	1	2	2	20	35	25	16	3	23	41
**Uni+Cor**		**0.1**	**Set1**	95	80	80	11	23	11	58	59	58	46	47	56	73
	
		**0.001**	**Set4**	55	43	39	0	0	0	0	15	7	4	3	4	11
	50% DE	**0.01**	**Set4**	65	52	54	0	0	0	18	30	24	13	14	23	43
		**0.1**	**Set4**	81	65	65	1	13	1	56	60	57	43	47	56	73

		**0.001**	**Set7**	0	100	100	1	67	40	100	100	100	1	0	100	100
	100% DE	**0.01**	**Set7**	0	100	100	1	67	40	100	100	100	10	1	100	100
**Bidir**		**0.1**	**Set7**	6	100	100	22	98	82	100	100	100	69	32	100	100
	
		**0.001**	**Set9**	0	84	82	0	7	2	32	95	93	0	0	86	93
	50% DE	**0.01**	**Set9**	0	95	95	0	7	2	93	100	100	1	0	100	100
		**0.1**	**Set9**	9	100	100	16	48	15	100	100	100	27	8	100	100

		**0.001**	**Set6**	0	84	84	0	11	8	5	28	17	6	1	15	25
	100% DE	**0.01**	**Set6**	0	88	89	0	11	8	39	50	43	17	8	43	61
**Bidir+Cor**		**0.1**	**Set6**	2	93	93	35	49	54	79	79	78	57	42	78	88
	
		**0.001**	**Set8**	0	63	62	0	2	1	3	30	14	0	0	13	23
	50% DE	**0.01**	**Set8**	0	75	76	0	2	1	36	50	42	3	0	40	58
		**0.1**	**Set8**	3	86	86	28	27	12	75	78	75	31	15	76	85

Each set of measurements presented was generated 100 times. The measurements in the table show, for each method, the number of detections of the different sets of measurements defined. The upper part of the table presents the results for unidirectional genesets only (all members over expressed) and the lower part concerns groups with a simulated mixed answer (over and under expression). The data related to H_0 _shows the number of genesets detected by chance (false positives).

Comparison of the results illustrates the effect of correlations between members of the sets. For each method tested, the presence of intra-set correlations reduces the performance of the analysis. For the simplest simulated sets (unidirectional), ANOVA-2 provides the best results, followed by FAERI (sets 1, 2 and 4) and GlobalTest, as evaluated using a gamma distribution or permutations (set 5).

Moreover, the ANOVA-2 and FAERI procedures are both associated with higher performance compared to all other methods based on the simplest simulation scenario (sets 1, 2 and 4). Indeed, FAERI detects set 1 in 62% of the cases, with a threshold of 0.001 and no false positives (set 3: 0%). At this threshold, FAERI is able to detect roughly 5 times more genesets than the best performing method currently available (SAMGS with q-value: 13%). As a comparison, SAMGS and GlobalTest require a threshold that is 100 times higher to detect only 60% of set 1. Comparison of sets 4 and 5 highlights the method's abilities to detect genesets where half of the members are not differentially expressed. In this case, FAERI detects 43% of the genesets, compared to 15% when applying GlobalTest with a gamma distribution. The ANOVA-2 and FAERI procedures thus appear to be the most appropriate to analyze unidirectional genesets.

The lower part of Table 4 concerns sets simulated with mixed differences in two directions (sets 6, 7, 8 and 9). The behavior of the methods differs: the ANOVA-2 and GSA methodologies (using the mean as the geneset statistic), as well as GSEA, are not appropriate to analyze such sets. For each bidirectional set tested, the FAERI methodology performs best, with the exception of set 9 where SAMGS provides better results for a threshold below 0.05 (values were checked for this threshold, but not included in Table 4). The presence of a correlation between the members reduces the performance, as shown for the unidirectional scenarios.

To ease interpretation of method's performances with regard to the mathematical structure of the genesets analyzed, Table 5 presents the ranking of the methods for each type of set simulated, using a threshold of 0.01 for the p-values. The statistic used was accuracy [the ratio of true positives and true negatives over the number of groups tested: accuracy = (TP/TN)/(P+N)]. The scores above 75% are shown in bold.

**Table 5:** Characterization of each method's performances

	a2.fixed	faeri. fixed.null	faeri. fixed.perms	GSA. mean*	GSA. absmean*	GSA. maxmean*	Globaltest. asymptotic	Globaltest. gamma	Globaltest. perm	Gsea. pval	Gsea. fdr	Samgs. pval
**100% uni**	**100%**	**99%**	**99%**	**57%**	62%	56%	**97%**	**99%**	**98%**	**99%**	**88%**	**99%**
**50% uni**	**94%**	**87%**	**89%**	**50%**	51%	50%	71%	9'%	92%	71%	62%	**90%**
**100%uni + cor**	**94%**	**84%**	**85%**	**50%**	51%	51%	60%	67%	62%	58%	51%	61%
**50%uni + cor**	**82%**	**76%**	**77%**	**50%**	50%	50%	59%	65%	62%	56%	57%	61%
**100% bidir**	50%	**100%**	**100%**	**50%**	**83%**	70%	**100%**	**100%**	**100%**	55%	50%	**100%**
**50% bidir**	50%	**97%**	**97%**	**50%**	53%	51%	**96%**	**100%**	**100%**	50%	50%	**100%**
**100% bidir + cor**	50%	**94%**	**94%**	**50%**	55%	54%	69%	75%	71%	58%	54%	**71%**
**50% bidir + cor**	50%	**87%**	**88%**	**50%**	51%	50%	68%	75%	71%	51%	50%	**70%**
**Mean**	71%	**90%**	**91%**	**51%**	57%	54%	**77%**	**84%**	**82%**	62%	58%	**81%**
**Rank**	7	2	1	12	10	11	6	3	4	8	9	5

Comparison of the number of true positives and true negatives to the total number of tests (VP+VN)/(P+N). For information, we also computed the mean value for the 8 types of groups studied. The significance threshold to select groups was set at 0.01. The highlighted (in bold) values are relative to methods obtaining a score of more than 75%. The four global methods are more appropriate for geneset analysis and the two-step methods show poor performance. Among the global methods, only ANOVA-2 and FAERI happen to be suited for unidirectional correlated genesets. FAERI and SAMGS are the only methods adapted for the study of bidirectional correlated genesets. Across all genesets tested, the methods giving the best results were: FAERI (perms), FAERI (null), SAMGS (*q-value*), GlobaTest (gamma), GlobaTest (perms), SAMGS (*p-value*), GlobalTest (asymptotic). FAERI is the only method suited for the study of all types of genesets tested.

Table 5 reveals that the best performing methods, independently of the structure of the set, are all based on a "global" strategy. The ranking of the methods is consistent with the models considered by these methods:

• ANOVA-2 is the most appropriate method for unidirectional groups;

• GlobalTest, which uses a statistic representative of response variability, is suited for the study of both uni- and bidirectional non correlated genesets;

• SAMGS, using an individual statistic, appears to be less appropriate for the analysis of correlated genesets, since this information is lost during the procedure;

• FAERI, developed to detect sets independently of their mathematical structure, is appropriate for each type of set tested;

• ANOVA-2 and FAERI provide an appropriate analysis of sets with correlated members, respectively using unidirectional and bidirectional definition of sets;

• Methods making use of a two-step strategy provide lower performance levels (GSA and GSEA).

#### Real data: study of hypoxia

Analysis of a genuine biological dataset offers the opportunity to qualitatively evaluate the results and provide a biological validation of geneset analysis methods through comparison of the results with current biological knowledge. We chose to use three datasets related to response under hypoxic conditions. The definition of the genesets relies on the MSIGDB databank (version 2.5), published by the authors of the GSEA methodology [25]. MSIGDB consists of several libraries of genesets (categories). The analysis reported below focuses on the C2.kegg category.

Table 6 lists the genesets detected by FAERI, evaluated on permutations, from analysis of the E-MEXP-445 dataset, described by Bosco et al. [39]. The detection threshold was set to 0.05 for the p-values (significant genesets). Each geneset selected was characterized by the p-values computed for several methods currently available. The last two columns provide the p-values characterizing the same genesets when the analysis was performed on 2 other datasets related to the same topic (GSE-1056 and GSE-4086).

**Table 6:** List of genesets in the C2.KEGG category detected significantly by the FAERI.perms method (detection threshold of 0.05 for the p-values)

	faeri. fixed.perms	faeri. fixed.null	a2.fixed	GSA. mean	GSA. absmean	GSA. maxmean	Globaltest. asymptocic	Globaltest. gamma	Globaltest. perm	Gsea. pval	Gsea. fdr	Samgs. pval	Samgs. qval	Gse1056	Gse4086
hsa00010_glycolysis_and_gluconeogenesis	**1.8E-03**	0.64	**1.2E-13**	0.96	0.95	0.98	**0.020**	**7.8E-03**	0.10	0.11	0.17	0.10	0.08	**0.047**	**8.0E-04**
hsa00020_citrate_circle	**6.9E-03**	0.38	**1.7E-05**	0.19	0.65	0.10	0.08	**0.035**	0.10	0.22	0.40	0.19	0.08	**0.020**	**1.9E-03**
hsa00030_pentose_phosphate_pathway	**8.4E-03**	0.41	**9.5E-04**	0.95	0.90	0.97	**0.024**	**8.8E-03**	0.10	0.23	0.28	0.10	0.08	**3.0E-03**	**0.019**
hsa00051_fructose_and_mannose_metabolism	**0.011**	0.97	**6.0E-04**	0.92	0.68	0.97	**0.043**	**0.014**	0.10	0.24	0.38	0.10	0.08	**0.025**	**3.9E-03**
hsa00100_biosynthesis_of_steroids	**4.0E-04**	0.94	**2.5E-04**	0.15	0.75	**0.0E+00**	0.14	0.10	0.30	0.11	0.32	0.10	0.08	**2.0E-04**	**1.0E-04**
hsa00190_oxidative_phosphorylation	**0.036**	0.99	**4.5E-07**	0.23	0.48	0.27	0.30	0.35	0.50	0.40	0.53	0.49	0.08	**0.0E+00**	**0.0E+00**
hsa00230_purine_metabolism	**0.047**	1.00	0.12	0.69	0.43	0.63	0.13	0.10	0.30	0.23	0.51	0.49	0.08	**7.0E-04**	**1.0E-04**
hsa00480_glutathione_metabolsim	**0.025**	1.00	**1.0E-03**	0.19	0.52	0.10	0.27	0.30	0.50	0.22	0.37	0.30	0.08	**9.0E-04**	**0.012**
hsa00511_n_glycan_degradation	**9.5E-03**	0.90	**0.020**	0.19	0.59	0.08	0.15	0.14	0.30	0.29	0.48	0.30	0.08	0.06	0.21
Hsa00530_aminosugars_metabolism	**0.031**	0.19	**0.040**	0.19	0.55	0.07	0.10	0.06	0.30	0.18	0.48	0.30	0.08	**0.026**	0.12
Hsa00620_pyruvate_metabolism	**0.011**	1.00	**2.6E-05**	0.09	0.68	**0.045**	0.06	**0.031**	0.10	0.11	0.19	0.10	0.08	**8.2E-03**	**5.0E-04**
Hsa00710_carbon_fixation	**0.022**	1.00	**3.3E-03**	0.95	0.90	0.97	**0.045**	**0.017**	0.10	0.23	0.35	0.10	0.08	**0.019**	**0.034**
Hsa00720_reductive_caboxylate_cycle	**0.031**	1.00	**4.2E-04**	0.10	0.75	**0.0E+00**	0.07	**0.034**	0.10	0.11	0.30	0.10	0.08	**4.6E-03**	**0.038**
Hsa00900_terpenoid_biosynthesis	**0.018**	**0.047**	**0.010**	0.25	0.77	**0.025**	0.06	**0.019**	0.10	0.48	0.63	0.10	0.08	0.07	**0.035**
Hsa01032_glycan_structures_degradation	**4.7E-03**	0.83	**3.9E-03**	0.19	0.55	0.13	0.14	0.13	0.30	0.10	0.43	0.40	0.08	0.05	0.08
Hsa01510_neurodegenerative_diseases	**0.010**	1.00	0.21	0.92	0.92	0.97	0.09	**0.043**	0.10	0.71	0.72	0.10	0.08	0.08	**9.0E-03**
Hsa03010_ribosome	**5.0E-04**	0.91	**1.1E-10**	0.78	0.57	0.86	0.24	0.26	0.40	0.11	0.22	0.29	0.08	0.27	**2.0E-04**
Hsa03320_ppar_signaling_pathway	**0.014**	1.00	**3.9E-03**	0.17	0.55	**0.035**	0.06	**0.039**	0.10	0.11	0.27	0.40	0.08	**0.017**	**6.9E-03**
Hsa04010_mapk_signaling_pathway	**0.020**	0.90	0.57	0.59	0.30	0.33	0.22	0.22	0.50	0.30	0.54	0.49	0.08	**0.0E+00**	**0.0E+00**
Hsa04130_snare_interactions_in_vesicular_transport	**8.6E-03**	0.97	0.11	0.18	0.56	0.11	0.16	0.10	0.40	0.61	0.71	0.40	0.08	0.48	**2.8E-03**
Hsa4150_mtor_signaling_pathway	**0.038**	0.23	**4.8E-03**	0.81	0.47	0.75	**0.039**	**0.010**	0.10	0.12	0.32	0.38	0.08	**0.017**	**0.039**
Hsa04210_apoptosis	**3.7E-03**	0.05	0.39	0.41	0.31	0.41	0.20	0.16	0.40	0.81	0.76	0.49	0.08	**1.0E-04**	**9.0E-04**
Hsa04370_vegf_signaling_pathway	**0.030**	0.08	**2.6E-03**	0.87	0.29	0.83	**0.047**	**0.017**	0.10	0.11	0.23	0.10	0.08	**7.0E-04**	**0.026**
Hsa04510_focal_adhesion	**0.035**	0.19	**0.025**	0.73	0.26	0.48	0.07	**0.034**	0.20	0.11	0.24	0.19	0.08	**4.3E-03**	**0.0E+00**
Hsa04620_toll_like_receptor_signaling_pathway	**5.7E-03**	**0.047**	**0.020**	0.61	0.47	0.66	**0.049**	**0.018**	0.10	0.11	0.44	0.10	0.08	**0.033**	**4.0E-04**
Hsa04650_natural_killer_cell_mediated_cytotoxicity	**0.016**	0.10	0.58	0.29	0.43	0.17	0.20	0.17	0.50	0.60	0.72	0.72	0.08	**7.0E-03**	**0.0E+00**
Hsa04660_t_cell_receptor_signaling_pathway	**0.036**	0.10	0.65	0.95	0.55	0.97	0.07	**0.025**	0.10	0.11	0.41	0.41	0.08	**1.6E-03**	**7.1E-03**
Hsa04662_b_cell_receptor_signaling_pathway	**1.5E-03**	0.97	0.78	0.40	0.48	0.13	0.06	**0.011**	0.10	0.53	0.63	0.63	0.08	**2.2E-03**	**2.0E-04**
Hsa04664_fc_epsilon_ri_signaling_pathway	**0.039**	0.97	0.05	0.91	0.55	0.97	**0.032**	**6.9E-03**	0.10	0.11	0.25	0.25	0.08	**8.9E-03**	**0.022**
Hsa04810_regulation_of_actin_cytoskeleton	**0.024**	0.52	**0.023**	0.30	0.40	0.38	0.29	0.32	0.50	0.42	0.52	0.52	0.08	**1.0E-04**	**0.0E+00**
Hsa05040_huntingtons_disease	**3.0E-04**	0.50	**6.6E-03**	0.78	0.95	0.98	0.13	0.06	0.10	0.29	0.45	0.45	0.08	0.83	**1.1E-03**
Hsa05150_cholera_infection	**0.019**	1.00	**2.9E-03**	0.37	0.73	0.11	**0.045**	**0.015**	0.10	0.37	0.51	0.51	0.08	0.12	**9.4E-03**
Hsa05120_epithelial_cell_signaling_in_helicobacter_pylorii_infection	**0.036**	1.00	**1.1E-03**	0.11	0.55	**0.045**	0.15	0.09	0.30	0.21	0.48	0.48	0.08	**1.3E-03**	**1.3E-03**
Hsa05130_pathogenic_escherishia_coli_infection_ehec	**0.047**	1.00	**0.044**	0.26	0.47	0.18	0.20	0.17	0.40	0.18	0.29	0.29	0.08	**4.9E-03**	**3.8E-03**
Hsa05131_pathogenic_escherishia_coli_infection_epec	**0.047**	1.00	**0.044**	0.26	0.47	0.18	0.20	0.17	0.40	0.18	0.29	0.29	0.08	**4.9E-03**	**3.8E-03**
Hsa05210_colorectal_cancer	**0.041**	**7.1E-03**	0.36	0.67	0.29	0.41	0.16	0.09	0.40	0.22	0.35	0.35	0.08	**0.011**	**8.8E-03**
Hsa05211_renal_cell_carcinoma	**0.017**	**7.1E-03**	**5.3E-04**	0.91	0.55	0.97	**0.036**	**9.9E-03**	0.10	0.12	0.24	0.24	0.08	**8.2E-03**	**0.021**
Hsa05212_pancreatic_cancer	**0.013**	**9.5E-03**	0.44	0.47	0.29	0.34	0.09	**0.041**	0.20	0.43	0.54	0.54	0.08	**4.3E-03**	**3.0E-03**
Hsa05216_thyroid_cancer	**4.0E-03**	0.86	0.91	0.53	0.55	0.20	0.17	0.15	0.30	0.30	0.54	0.54	0.08	**1.0E-03**	0.10
Hsa05219_bladder_cancer	**0.048**	0.47	0.60	0.57	0.55	0.61	0.11	0.08	0.30	0.91	0.80	0.80	0.08	**9.1E-03**	0.08
Hsa05220_chronic_myeloid_leukemia	**0.028**	1.00	0.73	0.37	0.31	0.41	0.15	0.09	0.40	0.61	0.65	0.65	0.08	**1.0E-04**	**7.9E-03**
Hsa05221_acute_myeloid_leukemia	**6.0E-04**	1.00	0.24	0.24	0.31	0.26	0.15	0.11	0.30	0.60	0.72	0.72	0.08	**0.0E+00**	**0.018**

For each geneset, the p-values obtained by each method are listed. The values in plain text are not significant. The p-values of the significant (0.05) and highly significant (scientific notation) genesets are shown in bold. The two last columns give the p-values obtained for datasets GSE-1056 and GSE-4086 by FAERI.perms, to illustrate the coherence of the results presented.

Table 6 shows that currently available methods fail to detect the genesets selected by FAERI (permutations). Most of the genesets selected by FAERI are also detected by at least one other global method, the ANOVA-2 or the GlobalTest. The two-step GSA.maxmean method seems to also be able to detect a few of the same sets. GSEA, SAMGS, GSA.mean and GSA.absmean do not find any significant genesets. Finally, the p-values obtained for datasets GSE1056 and GSE4086 are mostly significant or highly significant, validating the relevance of these genesets with regard to the biological question studied.

This list can be split into 3 categories. First, genesets concerning several metabolic pathways suggest metabolic adaptations occur as a cellular response to oxygen deprivation. Second, several genesets address signaling pathways and are known to be involved in the hypoxic stress response. Genesets of the third category are related to pathologies, mostly involving cancers, for which a hypoxic environment has been observed [52-54]. These results illustrate the ability of FAERI to detect groups known to be involved in hypoxic responses or hypoxia-related pathologies, and to detect those groups independently in 3 datasets related to hypoxia. In particular, we should point out the strong coherence between genesets detected by FAERI on a small number of replicates (3*3), either within the top list or between top lists associated with the three datasets.

These observations should be followed-up by a characterization of the genesets detected by the other methods. Table 7 lists the genesets detected with high significance (p-value < = 0.01) by at least one geneset analysis method (excluding FAERI), and compares the p-values assigned to these genesets by each of the methods tested (including FAERI). We observe that GSA.mean, GlobalTest (permutation), GSEA and SAMGS do not find any geneset to be highly significant. The sets detected by GlobalTest.gamma are also detected as significant by its equivalent based on an asymptotic distribution. Several genesets detected and considered to be significant by FAERI are related to hypoxia (hsa00010, hsa00030, hsa00720, hsa04664, hsa05211). Overall, the p-values attributed by FAERI to all the genesets detected by other methods are small, which confirms the ability of the method to assign lower scores to genesets related to hypoxia and detected by the other methods. Other genesets can be considered to be related to hypoxia, even indirectly (Cysteine metabolism, for example). The nature of these genesets suggests however that their relationship with hypoxia is of lesser importance than genesets known to be involved in the hypoxic response (listed in Table 6). FAERI assign a higher p-value to such sets (hsa00053, hsa00071, hsa00272, hsa00340, hsa00512, hsa00521). When the significance threshold is set to 0.05, the same conclusions can be made: GSEA and SAMGS fail to detect any significant geneset. On the contrary, GlobalTest gives a much higher number of genesets, and this list is coherent with FAERI (permutations) (data not shown).

**Table 7:** List of the genesets of the C2.KEGG category detected with a highly significant threshold (0.01) by the other methods

	Faeri. fixed.null	Faeri. fixed.perms	GSA. mean	GSA. absmean	GSA. maxmean	Globaltest. symptoti	Globaltest. gamma	Globaltest. permutations	Gsea. pval	Gsea. fdr	Samgs. pval	Samgs. qval
Hsa00010_glycolysis_and_neoglucogenesis	0.64	**1.8E-03**	0.96	0.95	0.98	**0.020**	**7.8E-03**	0.1	0.11	0.17	0.1	0.08
Hsa00030_pentose_phosphate_pathway	0.41	**8.4E-03**	0.95	0.9	0.97	**0.024**	**8.8E-03**	0.1	0.23	0.28	0.1	0.08
Hsa00052_galactose_metabolism	0.73	0.08	0.95	0.77	0.97	**0.018**	**8.3E-03**	0.1	0.11	0.13	0.1	0.08
Hsa00053_ascorbate_and_aldarate_metabolism	0.97	0.18	**0.0E+00**	0.72	0.06	0.16	0.16	0.3	0.11	0.42	0.19	0.08
Hsa00071_fatty_acid_metabolism	0.71	0.15	**0.0E+00**	0.52	**0.045**	0.2	0.18	0.5	0.11	0.3	0.49	0.08
Hsa00100_biosynthesis_of_steriods	0.94	**4.0E-04**	0.15	0.75	**0.0E+00**	0.14	0.1	0.3	0.11	0.32	0.1	0.08
Hsa00272_cysteine_metabolism	0.94	0.25	0.34	0.62	0.1	**0.028**	**6.7E-03**	0.1	0.21	0.46	0.19	0.08
Hsa00340_histidine_metabolism	1	0.17	**0.0E+00**	0.36	0.11	0.35	0.41	0.5	0.11	0.21	0.59	0.9
Hsa00500_starch_and_sucrose_metabolism	1	0.08	0.95	0.6	0.97	**0.020**	**6.7E-03**	0.1	0.11	0.18	0.1	0.08
Hsa00512_o_glycan_biosynthesis	0.2	0.22	**0.0E+00**	0.44	**0.0E+00**	0.36	0.42	0.5	0.64	0.71	0.1	0.08
Hsa00521_streptomycin_biosynthesis	0.94	0.1	0.95	0.96	0.97	**0.016**	**8.7E-03**	0.1	0.11	0.17	0.1	0.08
Hsa00640_propanoate_metabolism	0.92	0.06	0.15	0.6	**0.0E+00**	0.1	0.07	0.3	0.11	0.31	0.19	0.08
Hsa00720_reductive_carboxylate_pathway	1	**0.031**	0.1	0.75	**0.0E+00**	0.7	**0.034**	0.1	0.11	0.3	0.1	0.08
Hsa04664_fc_epsilon_ri_signaling_pathway	0.97	**0.039**	0.91	0.55	0.97	**0.032**	**6.9E-03**	0.1	0.11	0.25	0.1	0.08
Hsa0521_renal_cell_carcinoma	7.1E-03	**0.017**	0.91	0.55	0.97	**0.036**	**9.9E-03**	0.1	0.12	0.24	0.1	0.08

For each geneset, the p-values are given for each method we used.

As Table 6 illustrated a correlation between the results provided by FAERI on 3 related datasets, we compared each method's ability to discover common genesets from several related datasets. Table 8 lists the number of genesets detected by each method for each of the three datasets, as well as the number of common genesets between the datasets. The analysis was performed using the C2.kegg category, on the E-MEXP-445, GSE-1056 and GSE-4086 datasets. The thresholds were set to 0.01 and 0.05 for the p-values. The ANOVA-2 procedure, FAERI (permutations) and GlobalTest detected several genesets with a higher level of significance (lower p-value), while GSEA and SAMGS failed to detect any set, as mentioned previously. The GSE-4086 dataset contains only 4 (2vs2) replicates, and thus the analysis revealed a large rate of false positives. Therefore, we concentrated the analysis in Table 8 on datasets GSE-1056 and E-MEXP-445.

**Table 8:** Comparison of the number of groups detected in the C2.KEGG category and of the number of common detections in three datasets

0.010	A2. fixed	Faeri. fixed.null	Faeri. fixed.perms	GSA. mean*	GSA. absmean*	GSA. maxmean*	Globaltest. asymptotic	Globaltest. gamma	Globaltest. permutations	Gsea. pval	Gsea. fdr	Samgs. pval	Samgs. qval
Emexp445	62	7	14	14	5	19	0	8	0	0	0	0	0
GSE-1056	36	9	51	5	0	8	1	15	0	0	0	0	0
GSE-4086	93	89	94	49	38	62	0	0	0	0	0	NA	NA
Emexp445-GSE-1056	5	0	6	1	0	0	0	1	0	0	0	0	0
Emexp445-GSE-4086	24	5	9	8	0	11	0	0	0	0	0	NA	NA
GSE-1056-GSE-4086	21	7	37	4	0	4	0	0	0	0	0	NA	NA
Emexp445-GSE-1056-GSE-4086	4	0	3	1	0	0	0	0	0	0	0	NA	NA

0.050	A2. fixed	Faeri. fixed.null	Faeri. fixed.perms	GSA. mean*	GSA. absmean*	GSA. maxmean*	Globaltest. asymptotic	Globaltest. gamma	Globaltest. permutations	Gsea. pval	Gsea. fdr	Samgs. pval	Samgs. qval
Emexp445	62	18	42	14	5	23	26	45	0	0	0	0	0
GSE-1056	51	27	80	8	2	12	33	70	40	11	0	15	69
GSE-4086	119	119	142	49	38	62	32	185	0	0	0	NA	NA
Emexp445-GSE-1056	15	6	34	1	0	0	4	20	0	0	0	0	0
Emexp445-GSE-4086	48	14	37	8	0	13	13	44	0	0	0	NA	NA
GSE-1056-GSE-4086	31	23	71	4	1	6	7	69	0	0	0	NA	NA
Emexp445-GSE-1056-GSE-4086	13	4	31	1	0	0	2	20	0	0	0	NA	NA

The results are shown for each method, for significance thresholds of 0.05 and 0.01. The three datasets analyzed are E-MEXP-445, GSE-1056 and GSE-4086, and concern the same condition (oxygen deprivation). The first column contains the datasets analyzed. The first 3 rows of each table contain the top list obtained by each geneset analysis method. When several datasets are mentioned at the beginning of the row, the number of genesets in the other columns is the intersection of the results from different datasets

Setting a significance threshold of 1% for the p-values, the ranking of the methods detecting more genesets common to the two experiments is the following: FAERI.permutations (6 common genesets for at least 14 genesets detected), ANOVA-2 (5/35), GlobalTest.gamma (1/8) and GSA.mean (1/14). The other methods do not intersect. When the significance threshold is set to 5%, the ranking of the methods is the following: FAERI.permutations (34/42), GlobalTest.gamma (20/45), FAERI.null (6/18), ANOVA-2 (15/51), GlobalTest.asymptotic (4/26) and GSA.mean (1/8). The other methods do not intersect. These results suggest that the bidirectional genesets, detected by FAERI and GlobalTest, are an important part of the genesets simultaneously involved in both datasets, compared with the ANOVA-2 and GSA.mean procedures.

To quantify the abilities of the methods to provide the same result from different datasets, we propose to compute Pearson's coefficients of correlation on the ranks associated with the genesets analyzed, by pairwise comparison of the three datasets, for each method and each category of genesets defined (See Additional File 1, additional table 1).

For all the categories tested, the FAERI.permutations methodology is associated with a higher coefficient of correlation. Conversely, the ANOVA-2, GSEA and GSA.mean provide poorly correlated results. The coefficients of correlation obtained for the other methods are intermediary between these two extremes and depend on the geneset category.

## Conclusion

### Conclusions

Geneset differential expression analysis is a far more complex task than the individual analysis of expression changes. The diversity of the biological criteria involved and prior definition of the genesets has an impact on the mathematical properties of the expression subsets to be analyzed. Furthermore, the diversity of available analysis procedures, each based on specific strategies and null hypotheses, must address these properties. Thus, design of the analysis strategy is not that simple. Current methods involve over-representation analysis (ORA, not considered here), and functional class scoring (FCS). This last category of methods relies either on 2-step (post-hoc) or global strategies (using raw data).

In this paper, we address this question by considering the biological properties of genesets with regard to the underlying mathematical properties of the associated expression values. Focusing on expression levels, the direction of regulation and potential correlations between geneset members, we developed the FAERI methodology (*Functional Analysis: Evaluation of Response Intensities*). FAERI is a global methodology tailored from a 2-factor ANOVA procedure by a 2-step reduction of the data, and is evaluated with respect to the self-contained null-hypothesis (using label sampling or random data).

Evaluations performed on random data reveal the ability of each method to detect sets simulated according to 8 scenarios, relying on mathematical properties. The ANOVA-2 procedure performs best when analyzing unidirectional genesets and FAERI performs similarly. Both methods outperform each currently available method. When the definition of the sets involves members regulated in both directions, ANOVA-2 performance drops, as expected, and our proposed FAERI methodology outperforms all tested methods. Both methods provide an appropriate solution for the analysis of correlated data (exclusively on unidirectional genesets in the case of ANOVA-2). FAERI thus constitutes an improvement over all tested methods, and turns out to be the optimal method for testing question Q_0 _("Which known genesets are associated with different expression profiles under the two conditions compared?").

A real-world example of analysis is reported for three datasets on cellular response to hypoxia. The results obtained from analysis of the E-MEXP-445 dataset illustrate that FAERI (evaluated using permutations) is able to detect relevant results when a strict cut-off is used, compared to the other methodologies. The genesets detected by FAERI are related to three categories, respectively metabolic perturbations, hypoxia signaling/response, and hypoxia-related pathologies. The results of other global methods confirm these results, and post-hoc methods fail to detect any significant genesets. The results provided by FAERI from analysis of the two additional datasets reveal its ability to detect the same sets using several related datasets. Focusing on the top list obtained with several datasets, we showed that FAERI not only detects more sets, but also presents a larger intersection of the results.

We proposed to score this last assessment on the whole list of sets by computing the Pearson correlation coefficient on the ranked list of genesets, for each method, using pairwise comparisons between the three datasets. Among all methods tested, FAERI provides the best correlated results between related datasets, regardless of the source of geneset definition used (each category of the MSIGDB databank was used for this purpose). FAERI thus outperforms all methods tested for its ability to attribute similar scores to each geneset from several datasets.

Several recommendations emerge from this work. First, we suggest that differential expression analyses of microarray data be studied first by performing a geneset analysis with FAERI, and then that the results be further characterized using either an individual analysis or mathematical characterization of the sets detected. This approach eases interpretation of the results with regard to the correlation and direction of probeset-specific expression changes encountered for each geneset. Alternatively, genesets can be first classified with regard to the mathematical properties of the geneset expression values, and then the data can be analyzed using distinct methodologies (for example, applying the ANOVA-2 procedure on unidirectional sets).

### Perspectives

As a perspective for future work, we suggest to adapt FAERI so it would be able to test unequal sample datasets. Several adaptations to the ANOVA-2 procedure have been suggested to this end. To best adapt FAERI for this purpose, performance evaluations are required. Another interesting development would be to develop robust variants of FAERI based on the study of the impact of extreme values.

An additional comment may be formulated about the empirical definition of the direction from the difference in means, used in the second step of the FAERI procedure. Indeed, genes slightly differing in one direction may empirically seem to be involved in the opposite direction, as sampling a small number of replicates can lead to a smaller or larger distribution than the population. Thus, the directional reduction step may lead to an incorrect definition of the sign of the expression change with regards to the biological structure of the data. An alternative would be to characterize first the direction associated with each gene based on current knowledge of the biological processes. Similarly, another alternative adaptation of the procedure would be to determine which genes are associated with an expression change, and to use the directional reduction step only on those genes. However, the use of prior knowledge on the expression and directional changes for each gene would be associated with important biases: incorrect prior information would reduce the significance of the geneset analysis, and this bias is more probable for less-studied genesets/biological mechanisms. Thus, the evaluation of the null distribution with realistic background noise would be geneset specific, depending on the number of errors in the annotation. In the future, we also plan to develop a non-parametric equivalent to FAERI, by applying a rank-based multivariate analysis procedure after the 2-step reduction of data. At least three options may be considered for further developments: ranking expression values at the level of the probesets, at the level of the genesets, or at the level of the whole dataset.

The evaluation of significance may also lead to several adaptations. First, it would be interesting to add a third significance evaluation strategy based on simultaneous label sampling, where permutations would be performed without breaking the association with the cel file. Such a null distribution would provide an appropriate p-value for the evaluation of highly correlated genesets. However, the small number of available permutations would not allow for discrimination between genesets when the number of replicates is small. Both approaches introduce a bias during the p-value evaluation procedure: (i) the independent model assumes that the genes are not correlated, under-evaluating the p-value of co-occurring events; and (ii) the correlated model accordingly over-evaluates the p-value when the correlation between genes is not perfect. As the correlation pattern between genes is complex, the most appropriate p-value evaluation procedure should be somewhere in between the independent and correlated permutation models, with a geneset-specific balance between these two models.

Another interesting perspective for evaluation of the p-value would be to improve the model used to generate the null distribution from random data. By studying several datasets first using individual and geneset analyses, it would be possible to characterize the correlation between genes in several situations. Then the network of associations could be used to count the average number of connections, to catch patterns of association, etc. Using such a strategy, the evaluation of the null hypothesis would consist in generating random networks with similar patterns of association and similar distributions of connectivity, and to use these patterns using multivariate random data built from the simulated correlation matrices. As an extension of this model, it would also be possible to include a supplemental parameter during simulation of the null distribution: the correlation pattern between genes may differ between the two groups.

Performance evaluation of geneset analysis methods is not a trivial task, as each method is based on distinct null hypotheses. Previously published methods have been validated following several simulation schemes (using a limited number of scenarios) or running analyses on specific biological datasets (featuring more than 10 replicates for each tested condition). Nevertheless, those validations usually focused on the method developed by the authors, compared with only one method (usually GSEA), if any, and a limited number of genesets. Conversely, publication of individual analysis methods requires extensive validations on real-world, simulated and spike-in datasets. The validations reported here reproduce the simulation scheme used by Ackerman et al. (2009) as it represents the most comprehensive scenario we could find in the scientific literature (in a paper that compares the parameterization of current methods). The example analysis reported here was performed on small datasets (3 replicates associated with each condition), as this kind of dataset is far more frequent and more difficult to analyze. We hope that the validation strategy reported here will be reproduced by authors of future methodologies to compare their findings with current methods and to provide a comprehensive evaluation of their methods with regards to several biological scenarios and the underlying mathematical properties of the expression values.

In a previous work, we reported a new benchmarking strategy for individual expression analysis on real-world data involving several datasets [55]. In future work, this procedure will be tailored for a similar evaluation of geneset analysis methods with regard to the mathematical structure of the sets, as depicted in this paper on simulated data, and in agreement with the benchmarking study reported by Song et al. [56].

## Availability

FAERI and the ANOVA-2 procedure were implemented using R language, and the source code is included in PEGASE, an R package we designed for differential expression analysis [49]. URL: (http://urbm-cluster.urbm.fundp.ac.be/phoenix)

## Authors' contributions

FB scripted the FAERI methodology and performed the analysis presented here. BDM took part in the methodology performance assessment and creation of the methodology. AG took part in analysis and interpretation of the results. SD took part in analysis of the biological data. EB took part in scripting and automation. MP took part in interpretation and collection of the biological data. BDH took part in creation of the methodology and scripting. MD took part in the genesis of the methodology. ED coordinated the work and gave final approval for submission. All authors read and approved the final manuscript.

## Supplementary Material

Additional file 1 **Procedure section, describing the mathematical computing of FAERI**. Pearson's correlation coefficients computed on ranks, for each method between 3 datasets, for each geneset definition. Figure 1 in negative logarithmic scale. Histograms of the p-values under H0 for each method. Click here for file
