# Supplementary material for: Functional Analysis: Evaluation of Response Intensities - Tailoring ANOVA for Lists of Expression Subsets
Source: BMC Bioinformatics. 2010 Oct 13;11:510. doi: 10.1186/1471-2105-11-510 (PMC2964684; doi:10.1186/1471-2105-11-510)
Supplement: Additional file 1 — Procedure section, describing the mathematical computing of FAERI. Pearson's correlation coefficients computed on ranks, for each method between 3 datasets, for each geneset definition. Figure 1 in negative logarithmic scale. Histograms of the p-values under H0 for each method. [file 1471-2105-11-510-S1.DOC]

***Procedures***

This section presents the algorithm used for the FAERI methodology, as well as the two ways in which we evaluated the significance of the FAERI F* statistic.

**FAERI**

1. For each probeset, we computed the mean and standard deviation from the expression values associated with all samples hybridized (M_pool_ and S_pool_);
2. For each probeset, we used M_pool_ and S_pool_ to compute standardized expression values (Z-scores), using Equation 1;

| 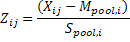 | Equation 1 |
| --- | --- |

where X_ij_ is the expression value associated with the i^th^ probeset in the j^th^ microchip, M_pool, i_ and S_pool, i_ are the means and standard deviations computed for probeset i from all experiments (step 1);

1. For each probeset, we computed the mean of the expression values associated with conditions A and B (M_A, i_ and M_B, i_) and then computed D_i_ = M_B, i_ – M_A, i_.
2. We used the vector of the difference of means (D_i_) to perform directional reduction of the data, thus producing a new matrix of expression scores using Equation 2.

| 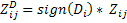 | Equation 2 |
| --- | --- |

where D_i_ = M_i_^B^ - M_i_^A^, the difference in means between conditions A and B for the i^th^ probeset, Z_ij_ = Z-score associated to the i^th^ probeset in the j^th^ microchip, and Z^D^_ij_ the directionaly reduced Z-score associated to the i^th^ probeset in the j^th^ microchip.

1. For each geneset, we computed the FAERI F* statistic associated with the phenotype in the same way as the Fisher F statistic of the classic ANOVA-2 procedure, using the probeset and phenotype as fixed factors (Error: Reference source not found to Error: Reference source not found).

**Significance evaluation**

Due to the directional reduction, the computed F* statistic is not distributed like the Fisher F statistic. In this paper, we propose to estimate the null distribution of F* using one of two evaluation procedures.

1. FAERI.null
2. Sample n*25,000 random values from a normal distribution, where n is equal to the total number of microarrays used (simulated data = 25,000 probesets).
3. Generate at least 1,000 genesets by sampling k simulated probesets from step A (where k is the geneset size).
4. Repeat step B for each geneset size.
5. Apply the FAERI methodology to the simulated data and compute the F*_0_ statistics for each simulated geneset.
6. FAERI.perms
7. Permute the data according to the self-contained null hypothesis (label sampling), independently for each probeset.
8. Analyze the permuted data using the FAERI methodology to compute the F*_perms_ statistics for each geneset.
9. Repeat steps A and B at least 1,000 times.

P-values can then be obtained by comparing the F* statistic of a geneset with the distribution of F*_0_ statistics with the same geneset size, or with the F*_perms_ computed from the label sampling procedure.

Supplementary table 1

**Suppl. Table 1 :** Pearson correlation coefficients, computed on ranks, for each method between 3 datasets, for each geneset definition. The analysis has been made from 3 datasets for all tested methods. Then, for each method, the p-value list is used to attribute a score to each group (rank). The correlation coefficient is computed on those ranks, starting from the 3 datasets. To reduce the size of the printed table, Pearson correlation coefficients computed on the ranks of the genesets were multiplied by 100 and rounded in order to get a 2-digit number.


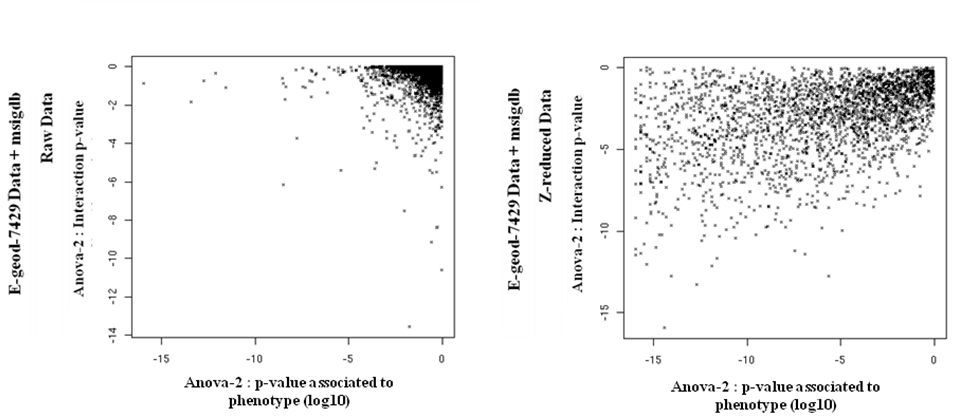


Figure 1 in negative logarithmic scale.


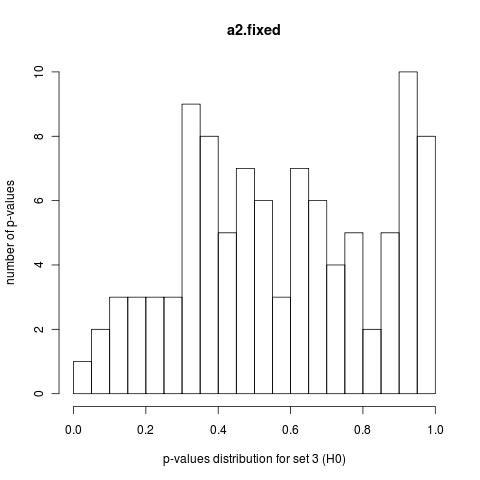

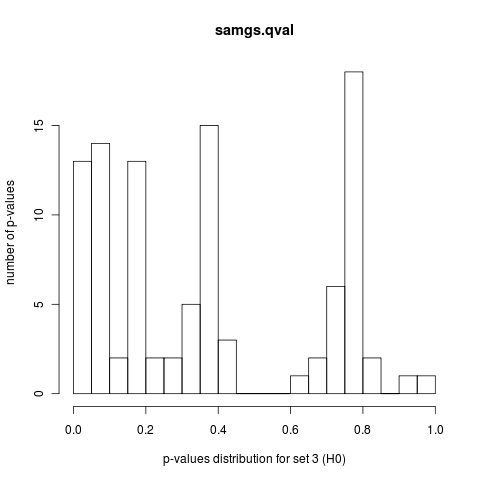

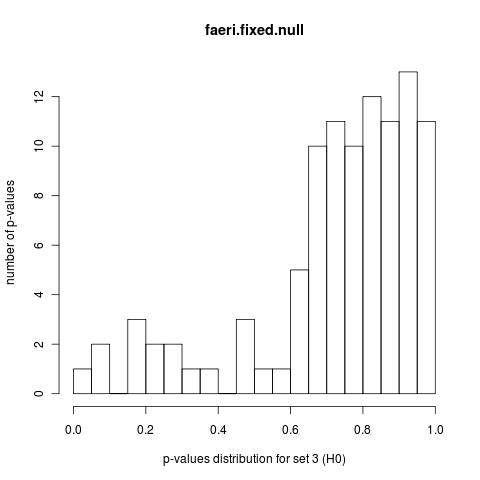

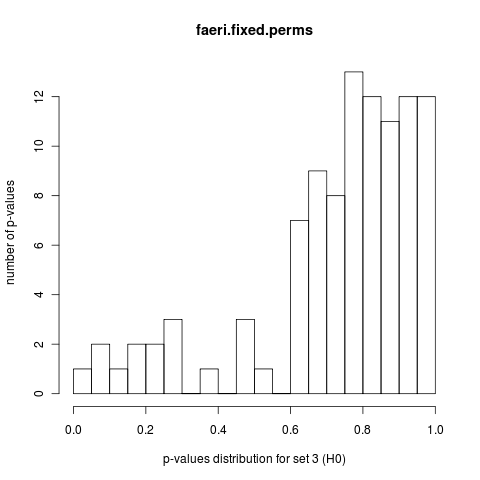

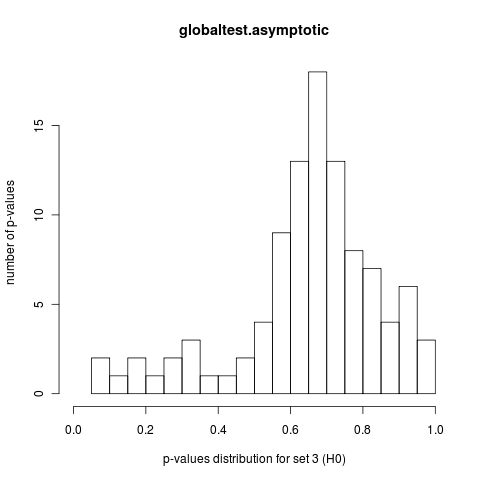

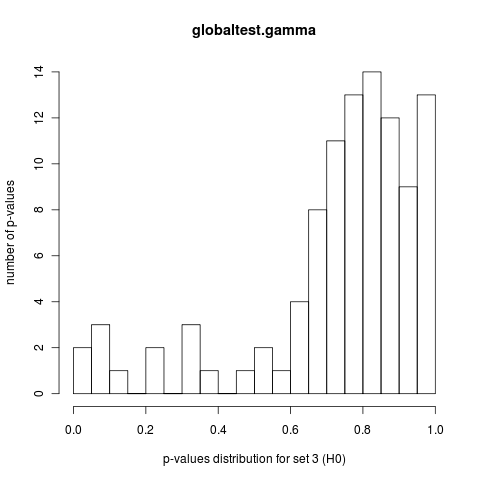

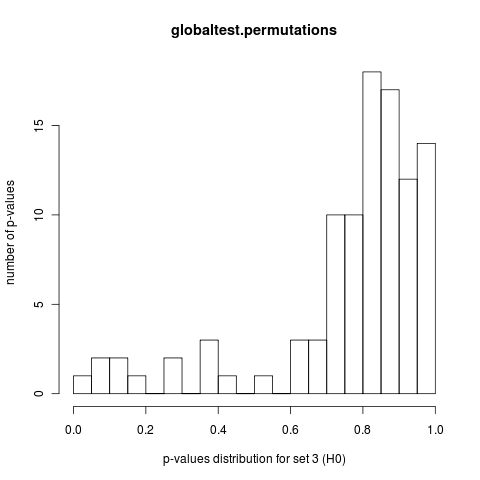

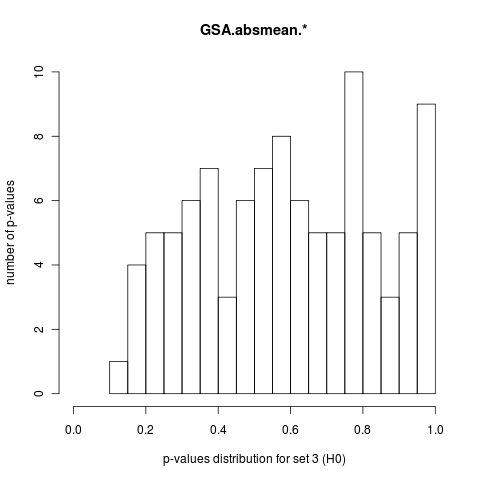

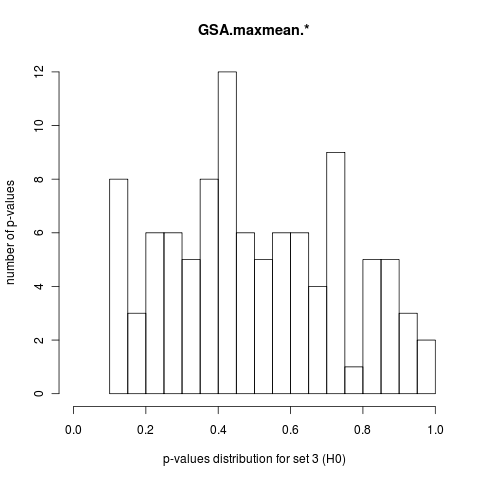

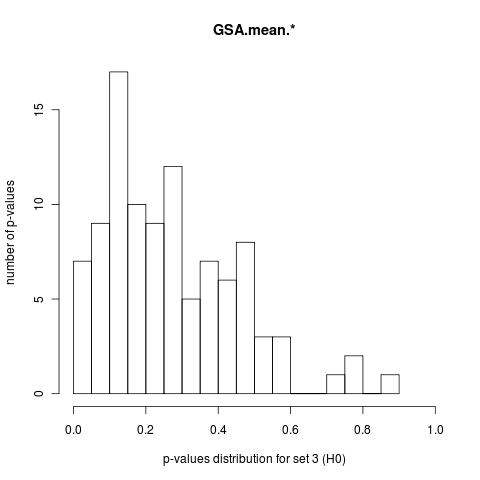

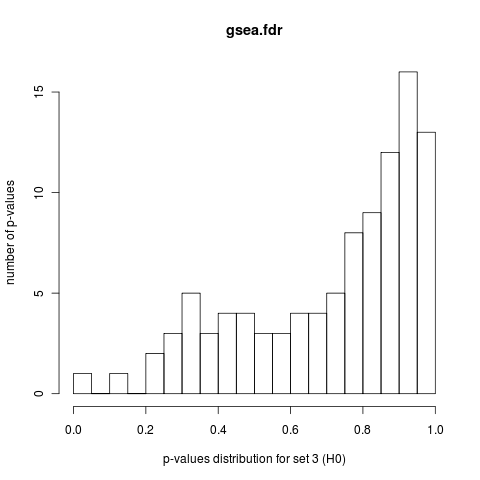

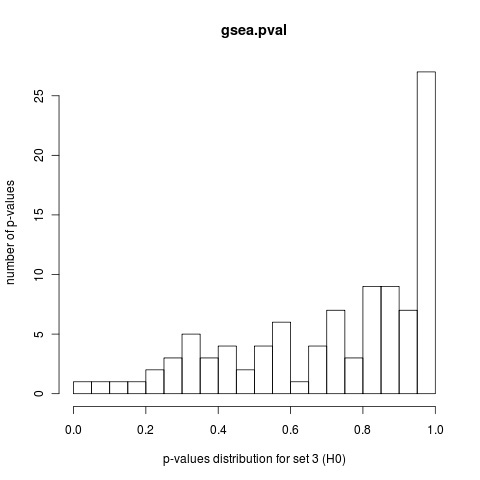

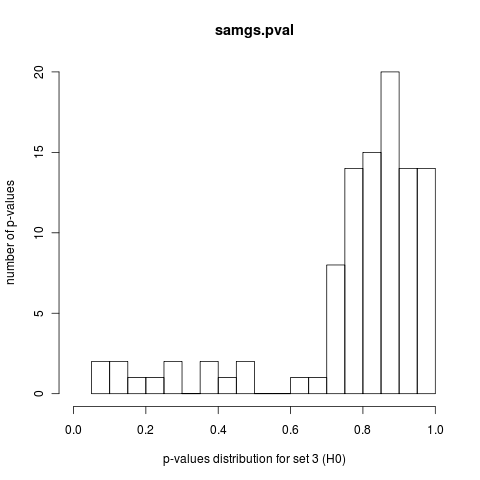


**Histograms of the p-values under H0** : Histograms of the p-values computed on simulations, under the null hypothesis. Individual values were generated without difference between conditions, and without correlation between genes. For each tested method, the distribution of the p-values is illustrated.
